# Assessment of Cataract Surgery Outcome Using the Modified Catquest Short-Form Instrument in China

**DOI:** 10.1371/journal.pone.0164182

**Published:** 2016-10-13

**Authors:** Jyoti Khadka, Jinhai Huang, Haisi Chen, Chengwei Chen, Rongrong Gao, Fangjun Bao, Sifang Zhang, Qinmei Wang, Konrad Pesudovs

**Affiliations:** 1 School of Ophthalmology and Optometry and Eye Hospital, Wenzhou Medical University, Wenzhou, Zhejiang, China; 2 Key Laboratory of Vision Science, Ministry of Health P.R. China, Wenzhou, Zhejiang, China; 3 Discipline of Optometry and Vision Science, Flinders University of South Australia, Bedford Park, South Australia, Australia; Save Sight Institute, AUSTRALIA

## Abstract

**Purpose:**

To assess cataract surgery outcome using the Rasch scaled Chinese version of the Catquest short-form.

**Methods:**

The Chinese translated and culturally adapted version of the Catquest-9SF was interviewer-administered to patients, pre and post cataract surgery. Rasch analysis was performed on the baseline data to revise the Catquest. For the surgical outcome assessment, we stacked pre- and post-surgical Catquest data to demonstrate improvement in visual function scores and responsiveness of the instrument to cataract surgery.

**Results:**

A total of 247 cataract patients (median age, 70 yrs; male 51.0%) completed the Catquest 9SF at baseline.The Catquest-9SF possessed adequate measurement precision of 2.15. No disordering of response categories were observed and all the items perfectly fit to the Rasch model except item 7 (outfit >1.5). A slight reduction in precision was observed after removing misfitting item 7 (Catquest-8SF-CN), but the precision value was well above the acceptable value of 2.00. Notably, the instrument was well targeted (mean person location 0.30), demonstrated no evidence of multidimensionality and DIF. At 12 months post-surgery, 74 (30%) patients came for follow-up and completed the Catquest. There was a significant improvement in the Catquest scores post cataract surgery with a considerably large effect size.

**Conclusion:**

The Catquest-8SF-CN demonstrated promising Rasch based psychometric properties and was highly responsive to cataract surgery.

## Introduction

Cataract is the leading cause of blindness and vision impairment in the world.[1–3] China not only boasts the largest population in the world but also the largest population with cataract (estimated 2.5 million with cataract and incidence of 400 thousand per year).[4–6] Even though, a simple surgical procedure can restore vision, the discord that exists between the number of people with cataract and the surgical rate has created a huge backlog of patients needing cataract surgery in China.[7,8] Therefore, a large population has to live with treatable vision loss and the consequent low quality of life (QoL). It has been now well accepted that the overall QoL impacts of cataract and improvement after cataract surgery cannot be assessed just by clinical visual function assessments alone such as visual acuity.[9,10] More recently, patient-reported outcomes (PROs) instruments are increasingly accepted as necessary clinical and research outcome measures including in cataract surgery outcomes.[11,12] Moreover, the U.S. Food and Drug Administration (FDA) has regarded PROs as an important index of primary endpoint in health research owing to the notion that such research ultimately guide patient care.[13] Over the past few decades, many cataract-specific PRO instruments have been developed and validated for use in the developed countries.[14–18] Among the existing cataract-specific PRO instruments, the Catquest-9SF is considered as one of the best cataract-specific instruments in terms of its sound psychometric properties, low respondent burden and responsiveness to cataract surgery.[10,15,18,19]

The Catquest-9SF has recently assessed for its psychometric properties using Rasch analysis in a Chinese population [20], albeit, it has not yet been tested for its responsiveness to cataract surgery in China. Therefore, we aimed to assess cataract surgery outcome using the Rasch scaled Chinese version of the Catquest. Furthermore, we also aimed to optimize any deficiency in psychometric properties of the Catquest-9SF using Rasch analysis.

## Patients and Methods

The patients were recruited from the Eye Hospital of Wenzhou Medical University and completed the PRO instrument by face–to-face interviews before and 12 months after surgery at the hospital. The eligibility criteria were Chinese adults over 18 years old, who had a definitive diagnosis of cataract. Patients with cataract as their primary diagnosis and who attended our hospital for surgery were included. Exclusion criteria were those with a significant hearing or cognitive impairment that might have limited their ability to respond to a PRO instrument, had other significant co-existing eye diseases and had severe co-existing systemic comorbidity which might have influenced their visual function. Patient with a co-existing ocular morbidity that might have contributed to a significant loss of vision and their self-reported visual function were also excluded from the study. For this, we excluded patients with significant corneal disease (corneal leukemia, pterygium invade the central corneal and cover the pupil area), glaucoma (with VA <6/60 and visual field loss within the central 10, or the MD of the binocular VF loss <-12dB), diabetic retinopathy (proliferative diabetic retinopathy, moderate and sever degrees of diabetic macular edema), macular diseases (geographic age-related macular degeneration with VA<6/60 and all exudative age-related macular degeneration, macular hole stage II or Higher) and other retinal disease (with VA<6/60 of retinal vasculitis, retinal detachment, retinal pigmentosa).[19] This study was approved by the review board of Eye Hospital of Wenzhou Medical University, Wenzhou, China and followed the tenets of the Declaration of Helsinki. All participants provided written informed consent after the nature of the study had been explained to them.[21]

### The Catquest-9SF

The 9-item Catquest short-form questionnaire (Catquest-9SF) was developed from the original long version Catquest questionnaire using Rasch analysis.[15] The Catquest-9SF demonstrated promising psychometric properties and was found to be highly responsive to measure cataract surgical outcomes.[10,15,19,22] It has also demonstrated good Rasch based psychometric properties when tested in Australian and Chinese cataract population, albeit its responsiveness to surgery was not tested in those two settings. The Catquest-9SF consists of 3 types of items (9 items in total); a global daily life difficulty item (item 1), a global vision satisfaction item (item 2) and a group of 7-items referring to difficulties in performing day-to-day activities, e.g. reading text, recognizing people's face, seeing to walk on uneven road (item3-9). The response categories for question 1, 3–9 are “yes, very great problems”, “yes, great problems”, “yes, slight problems”, “no, no problems”, and “cannot determine”. The response category for question 2 is “very unsatisfied”, “fairly unsatisfied”, “fairly satisfied”, “very satisfied”, or “cannot determine”.

In this study, the English version of the Catquest-9SF was translated into Chinese (into Mandarin) independently by two competent bi-linguistics and experienced translators. The two versions were reviewed and adjusted by a panel of experts to form the first draft of the Catquest-CN (where “CN’ stands for Chinese version). A third bi-linguistic translator, who was not involved in the previous translation, then re-translated the Chinese version back into English. Any discrepancy between the original English version and the English back translated version were identified, reviewed and revised by the panel for semantic equivalence to form the second draft of the instrument. The Chinese version was then recited to 20 cataract patients to specifically test items comprehension and cultural sensitivity. Further revisions on each item wordings were carried out on the basis of patients’ feedback when it was deemed necessary by the panel to match the Chinese socio-cultural norms to enhance comprehension of the items. This exercise helped to streamline comprehension and cultural modification of the items whilst ensuring semantic equivalence to the English version (Table 1).

**Table 1:** Item content of the English version and Chinese version.

Item	English version	Chinese version
1	Daily-life activities in general	Daily-life activities in general
2	Satisfaction with vision	Satisfaction with vision
3	Reading text in the daily paper	Reading text in the newspaper
4	Recognize the faces of people you come across	Recognizing faces of people you meet
5	See prices when shopping	Seeing prices of goods when shopping
6	Seeing to walk on uneven ground	Seeing to walk on uneven ground
7	See to do handwork, woodworking, etc.	Seeing to do delicate work (needlework, handwork, carpentry, etc.)
8	Reading text on TV	Reading text on television
9	See to carry on an activity/hobby you are interested in	Seeing to carry out a preferred hobby

Even though, the Catquest-9SF was translated into Mandarin, it was interviewer administered in local dialects (e.g. Cantonese, Wenzhounese) if the participants did not understand Mandarin. In order to minimize the influence of different dialects on the validity of the Catquest 9SF-CN in this study, Mandarin was used priory to other dialects when investigating. Utmost caution was practiced when administering the instrument to patients who spoke different dialects. Basically, our trained staff communicated in the interviewees’ dialect/s while administering the instrument face-to-face if Mandarin could not be understood well. During this we also made sure that the verbal translation in other dialects was in accordance with the original meaning of the instrument both in its meaning and phraseology.

We used baseline data of the participants (psychometric assessment group) to test and improve the psychometric properties of the Catquest questionnaire. We only used pre- and post-operative data of those who followed-up for outcome assessments (Outcomes group). The outcomes group was divided into first-eye, both-eyes and second-eye surgery groups for the sub-group analysis. All the second eye surgery patients had undergone their first eye cataract surgery at least 6 months ago.

### Rasch analysis

The Rasch model is a probabilistic mathematical model that estimates person ability, item difficulty on a continuum logit measurement, a person with a higher ability and an item with greater difficulty is expressed on the negative side of the logit sale for this study. The assessment of Rasch model includes category threshold order, precision, item fit statistics, unidimensionality, targeting and differential item functioning (DIF).[23] Due to the polarity of the response categories, a higher negative logit value indicates better score and vice versa.

#### Category threshold order

The category threshold ordering is a very important parameter to demonstrate the usage of response categories is in an orderly fashion by the respondents. Disorder categories occur when the categories are hard for participants to discriminate, categories are underused, or the definitions of categories are unclear; the solution is to collapse the categories until all the categories are ordered.[24,25]The ordering of response categories is analyzed at first during psychometric assessments of an instrument. This is because, ordering or disordering of response categories have greater influences on other metric properties of items than vice versa.[26–28]

#### Measurement precision

The person separate index (PSI) is the measure of discriminant capacity of an instrument to identify people having different levels of underlying traits being measured. A PSI ≥ 2.0 indicates that the PRO instrument can discriminate people with at least 3 levels of abilities (for example: mild, moderate and severe). The higher is the PSI value; the better is the overall precision of the PRO instrument.[14,15,23,24]

#### Item fit statistics

The item fit statistics indicate the extent to which the data match the Rasch model. It is assessed by two fit statistics: infit and outfit mean squares (MNSQ), the values of which are expected to be 1, the strict fit range are 0.7–1.3.[15] However, many researchers have adopted a more lenient MNSQ range from 0.50 to 1.50.[29–31] In this study, we have also adopted the lenient criteria of item fitting. The infit statistic is more sensitive to the data when the item difficulties match the person ability well, thus considered being more informative fit statistic.[14,15,22,23,32] A PRO instrument with perfectly fitting items is more likely to be unidimensional.

#### Unidimensionality

In addition to the item fit statistics, the principal components analysis (PCA) is a more definitive test to assess unidimensionality. When the level of variance explained by the raw data >60%, and the first contrast has an eigenvalue of <2.0, the scale is considered unidimensional.[14,15,22–24]

#### Targeting

Targeting indicates how well the items difficulty matches the person ability. The difference of the item and the person mean values indicates targeting; a perfect targeting exists when the difference between two means is zero and a value >1.0 indicates poor targeting.[14,15,22–24]

#### Differential Item Functioning (DIF)

DIF occurs when subgroups of persons within the same study cohort with comparable ability answer differently to an item. For this study, we assess DIF of each item by age (≤60, >60), gender (male, female), systemic and ocular comorbidities (present, absent), cataract status (first eye, second eye). DIF magnitude of <0.5 logit is considered small or absent, 0.50 to 1.0 logit is minimal, and >1.0 logit is notable.[15,25]

#### Validity

The construct validity of the instrument was assessed by item separation index (ISI) and item separation reliability (ISR) values. An ISI of ≥ 3 or more and ISR of ≥ 0.9 indicate that the study population size is satisfactorily large enough to draw strong inference about the items hierarch on a difficulty continuum scale.

The correlation between the instrument scores and visual acuity is typically used to indicate criterion validity, which refers to the assessment of whether the instrument measures what it intends to measure. The correlation between the Catquest-CN and baseline visual acuity was calculated using the Spearman’s correlation co-efficient to test the validity of the instrument. The co-efficient of <0.2 is considered weak, which means the two data which are hypothesized to be related are indeed not well related at all. A value between 0.2 and 0.8 is considered moderate, which is a mostly expected result in a questionnaire study because it suggests the two measures are related and also provide different information. A value of >0.8 is considered high correlation, suggesting that little or no additional information could be gained by using the two measures simultaneously.[19,25]

### Outcome assessment: sample size and responsiveness

The minimum sample size required for the surgical outcome assessment was calculated based on the previous study that used the Catquest-9SF in a Swedish population.[10,15] We used the following formula to calculate the sample size in each of the pre and post-operative group.[33,34]

$$n=\frac{2[{\left(a+b\right)}^{2}\;\sigma^{2}]}{{\left(\mu_{1}+\mu_{2}\right)}^{2}}$$

Where, n = the sample size in each of the groups; μ_1_ = mean pre-surgical Catquest score; μ_2_ = mean post-surgical Catquest score; σ = population variance, a = 1.96 for the significant level alpha at 0.05; b = 0,842 for beta chosen at 0.20 (i.e. power = 80%)

Using the findings of the Swedish study (the mean Catquest scores for pre- and post-surgery were -0.32 and -3.21 logits respectively with an SD of 2.32), the minimum required sample size of 9 in each pre and post-surgery groups (i.e. n = 18 in a group) was estimated.

The responsiveness of the Catquest was assessed by calculating an effect size for all those who came for follow-up (overall group) and 5 other sub-groups (i.e. 1^st^ eye surgery, both eyes surgery, second eye surgery, with ocular comorbidity and without ocular co-morbidity). The effect size (ES) was calculated to assess responsiveness of the Chinese Catquest to cataract surgery. The ES is the mean change in Catquest scores divided by the pooled standard deviation of the pre-surgical and post-surgical scores. [35] The pooled standard deviation is weighted to each group’s standard deviation by its sample size and is calculated usingthe following formula.

$${SD}^{*}{}_{\;pooled}=\sqrt{\frac{(n_{1}-1){SD}_{1}^{2}+(n_{2}-1){SD}_{2}^{2}}{n_{1}+n_{2}-2}}$$

The ES of 0 to 0.20, 0.20 to 0.80 and more than 0.8 were considered small, medium and large, respectively. The ES was also considered very large if the 95% confidence interval (CI) around the ES is more than 1.0.[10]

### Statistical analysis

For the psychometric assessment of the Catquest, Rasch analysis was performed using the baseline data as a group analysis with 1 rating scale model per question format using Winsteps (version 3.91.0 Winsteps, Beaverton, Oregon, USA). For the outcome assessment, we pooled pre- and post-surgical data to run a single Rasch analysis with post-surgical data considered as “new patients.” [36,37] Then, pre and post-surgical Catquest scores were obtained for each patient. The stacking method for this analysis was used because it creates a measure in the same frame of reference scale. This allows more accurate comparison of the effect the cataract surgery between two time-points. For sub-group analysis, patients were stratified into three groups: first eye only, both eyes and second eye only. A one-way between-groups analysis of variance was conducted to explore the difference in pre- and post-operative visual function scores measured by the Catquest. If the analysis showed a statistical significance a post hoc comparison between the groups was also carried out using the Tukey HSD test.

We also calculated the effect size for the overall and other five subgroups (first eye, both eyes, second eyes, with and without ocular co-morbidities). We also assessed outcomes by visual acuity (VA). A Kruskal-Wallis test was carried out to explore the difference in pre-surgical VA among the three sub-groups. We analyzed all the data using SPSS software (version 20.0, SPSS, Inc.). Mann-Whiteny U, Wilcoxon Signed Rank test and Spearman rank correlation were used if 1 datum or both data were not distributed normally. An independent-Sample T Test was used to compare improvement in visual function (the Catquest scores were normally distributed) before and after surgery. A P value less than 0.05 was considered statistically significant. The effect size and the 95% CI were calculated using Centre for Evaluation & Monitoring online effect size calculator (http://www.cem.org/effect-size-calculator). We also performed post-hoc power calculation using an online calculator available from http://www.danielsoper.com/statcalc.[38]

## Results

A total of 247 cataract patients completed the Catquest-9SF a day before the cataract surgery (Table 2). There were slightly fewer females (49%), the median age was 70 years, most of the patients were waiting for the first eye surgery (86.2%; first and one eye, 49.8%; first and both eyes, 36.4%), over a half of the participants had either ocular or systemic co-morbidities and the majority had poor educational background (only 12.2% attended senior middle school and university). Out of 247, only 74 (30%) patients came for 12-month follow-up and completed the Catqusest-9SF. Out of 74 patients, 32, 28 and 14 patients had undergone first-eye, both eyes and second eye cataract surgery respectively (Table 2).

**Table 2:** Baseline demographic characteristics of the participants.

Characteristics	Psychometric assessment	Outcome assessment
	(n = 247)	(n = 74)
Median age years (IQR; range)	70 (63to77; 36 to 92)	69 (62 to 75;40 to 88)
Sex, n (%)		
Female	122 (49)	41 (55.4)
Surgical eye/s, n (%)		
First eye surgery	123 (49.8)	32 (48.6%)
Both eyes surgery	90 (36.4)	28 (40.5%)
Second eye surgery	34 (13.8)	14 (10.8%)
Pre-operative VA LogMAR median, (IQR)		
First eye (Operated)	0.82 (0.30 to 1.70)	0.92 (0.68 to 1.50)
Second eye (Operated)	0.50 (0.30 to 1.60)	0.70 (0.32 to 0.98)
Both eyes (Worse eye)	0.92 (0.60 to 1.40)	0.96(0.30 to 1.20)
Both eye (Better eye)	0.60 (0.4 to 0.94)	0.60(0.30 to 0.84)
Post- operative VA LogMAR median, (IQR)		
First eye (Operated)		0.10 (0.07 to 0.20)
Second eye (Operated)		0.20 (0.03 to 0.40)
Both eyes (Worse eye)		0.15 (0.10 to 0.40)
Both eye (Better eye)		0.10 (0.07 to 0.20)
Ocular comorbidity*, n(%)	121 (49.0)	42 (56.7)
Glaucoma	10 (8.2)	3 (7.1)
AMD	4 (3.3)	2 (4.7)
DR	9 (7.4)	3 (7.1)
Pathological myopia	39 (32.2)	14 (33.3)
Corneal disorders	8 (6.6)	0
Others	91 (75.2)	20 (47.6)
Systemic comorbidity**, n (%)	170 (68.8)	52 (71.1)
Hypertension	116 (68.2)	37 (71)
Diabetes	67 (39.4)	21 (40.3)
Others	74 (43.5)	17 (32.7)
Educational status, n (%)		
Illiterate	62 (25.1)	15 (20.3)
Primary school	90 (36.4)	25 (33.8)
Junior middle school	65 (26.3)	20 (27.0)
Senior middle school	15 (6.1)	8 (10.8)
University	15 (6.1)	6 (8.1)

VA = Visual acuity, LP = light perception, IQR = interquartile range.

*Includes diabetic retinopathy (DR), glaucoma, age-related macular degeneration (AMD), corneal disorders (corneal macula, corneal dystrophies), pathological myopia, and other eye diseases (pterygium, vein occlusion, uveitis, epiretinal membrane etc).

** Percentages of co-morbidities add more than the total sum; as some ocular and systemic conditions co-exist.

The Catquest-8SF-CN had demonstrated similar Rasch-based psychometric properties but better targeting to the population ability than in other studies (Table 3).

**Table 3:** Rasch based parameters of the Catquest-9SF instrument developed in different versions.

Parameter	Swedish version (Lundstrom et al)	German version (Harrer et al)	Australian version (Gothwal et al)	Current study	
	9SF	9SF	9SF	9SF	8SF*
Number of items	9	9	9	9	8
Measurement precision	2.58	2.74	2.28	2.15	2.09
Misfitting items	0	0	0	1	0
Mean person location	-0.22	-1.36	-0.86	0.64	0.50
PCA (eigenvalue 1^st^ contrast)	1.8	NA	1.7	1.7	1.7

NA = not available

*In this version (Catquest-8SF) item 7 was deleted

### Category threshold order

**Fig 1:**
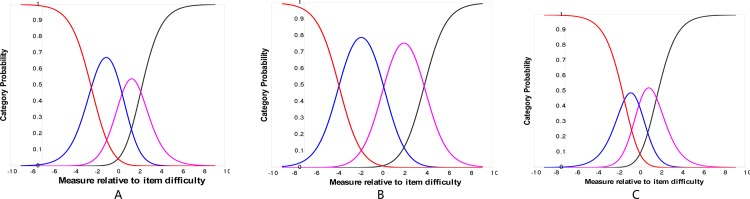
(A) Category probability curve for the “global question about any difficulties in daily life” item. (B) Category probability curves for the “satisfaction in vision” item. (C) Category probability curves for the 7 visual disability items.

The response categories were ordered (Fig 1A–1C) indicating that the response categories were understood and discriminated as separate entity by the participants across three different question formats.

### Item fit

All the items except item 7 demonstrated good fit. Item 7 of “Seeing to do delicate work (needlework, handwork, carpentry, etc.)” had outfit value of 1.74. Therefore, this item was deleted from the scale. After the deletion, the remaining items (8SF) fit perfectly well to the Rasch model (Table 4), the measurement precision stayed high and the response categories remained ordered.

**Table 4:** Item measure and fit Indices of the Catquest-9SF Scale.

Item	Location ±(Standard Error)	Infit MNSQ	Outfit MNSQ
Two global assessment items			
1. Daily-life activities in general	-0.20±0.11	0.76	0.73
2. Satisfaction with vision	-2.71±0.13	0.83	0.79
3. Reading text in the newspaper	-0.55±0.13	1.32	1.20
4. Recognizing faces of people you meet	1.15±0.10	1.11	1.13
5. Seeing prices of goods when shopping	-0.02±0.12	0.80	0.76
6. Seeing to walk on uneven ground	0.97±0.10	0.98	1.07
8. Reading text on television	-0.01±0.10	0.90	1.10
9. Seeing to carry out a preferred hobby	1.37±0.11	1.43	1.42

MNSQ = mean square

### Measurement precision

The precision of 9SF was 2.15, after deleting item 7, the PSI slightly dropped to 2.09 which was still higher than minimum acceptable precision value.

### Targeting

**Fig 2:**
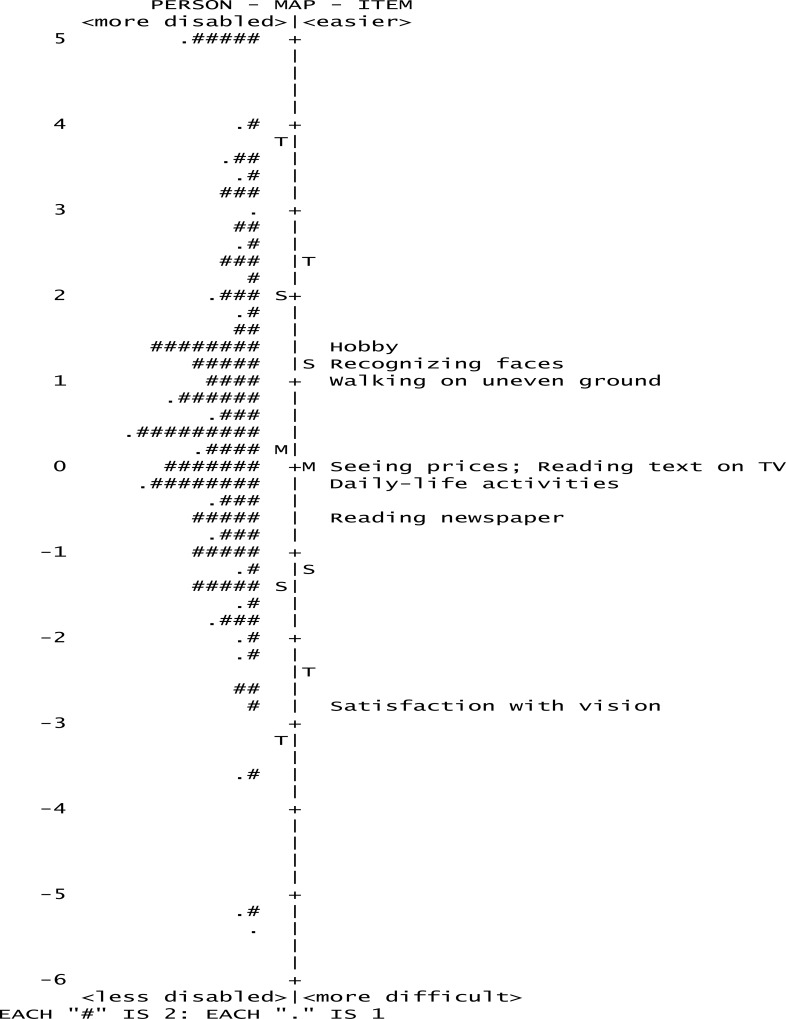
Person–item map of the 8-item Catquest-8SF for the cataract group showing the distribution of Rasch calibrated participant scores (left) and item locations (right). The items are well targeted to the patients as illustrated by the matching of the distributions. (M = mean, S = 1 standard deviation, T = 2 standard deviations. Each "#" is 2. Each "." is 1.)

The mean person location of 9SF was 0.64, after deleting the item 7, the targeting improved (Table 3). However, mean person location for 8SF was still <0.50 (Fig 2), which signifies that the instrument had excellent targeting to our study population.

### Unidimensionality

The principal components analysis showed 60.8% and 61.4% of the variance of the amount of raw variance was explained by the measure for empirical calculation and by the model respectively. The unexplained variance explained by the first contrast had the eigenvalue of 1.7, indicating the assumption of unidimensionality was met.

### DIF

None of the 8 items showed DIF by age, gender, co-morbidities (ocular and systemic), pre and post-surgical groups and unilateral vs bilateral cataract r surgery groups.

### Validity assessment

The item separation index and reliability were 10.11 and 0.99, respectively. These indicate construct validity of the Catquest-8SF-CN. Given the skewed distribution of visual acuity, Spearman’s rank test was used to calculate the correlation between the Catquest-8SF scores and the visual acuity. A moderate correlation was observed for both the eyes (r = 0.490, p<0.0001), while the correlation was stronger between the Catquest-8SF and the better eye visual acuity (r = 0.489, p, 0.0001) than the worse one (r = 0.249, p<0.0001) suggesting perhaps the better eye visual acuity was nearly equivalent to the binocular visual acuity. This indicates criterion validity of the instrument.

### Outcome assessments

There was no statistically significance difference in pre-operative VA in better (Chi-square (2,n = 74) = 5.27, p = 0.07) and worse eyes (Chi-Square (2, n = 74) = 1.03, p = 0.5) among the three groups. However, there was a statistically significant improvement in VA in the operated eye first-eye surgery group (z = -4.920, p = 0.001), second-eye surgery group (z = -2.4, p = 0.017) and for the both-eyes surgery group (better eye z = -4.45, p<0.001; worse eye z = -4, 46, p<0.001).

Preoperatively, there was a significant difference in mean visual function measured by the Catquest between the 3 groups (F (2, 71) = 3.8, p = 0.03) and Tukey HSD post hoc test showed that this was due to difference between the first-eye and both eye surgery groups (mean difference = -1.10, p = 0.02). Postoperatively, the mean visual function improved significantly in all the three groups (Table 5). However, the improvement in visual function was not statistically different among these 3 groups (F (2, 71) = 0.2, p = 0.82).

**Table 5:** The pre- and post-surgery Catquest scores, change in scores and the effect size.

	
Number	Pre-surgery* (SD)	Post-surgery* (SD)	Change (SD)	t #	Effect size (95% CI)	Post-hoc power calculation
Overall(74)	0.32 (1.69)	-2.75 (2.17)	3.07 (0.32)	9.6	1.58 (1.21to1.94)	0.99
First eye (32)	-0.10 (1.38)	-2.92 (2.14)	2.81 (0.45)	6.2	1.57 (1.00 to 2.11)	0.84
Both eyes (28)	0.80 (1.40)	-2.67 (2.42)	3.48 (0.53)	6.6	2.20 (1.51 to 2.83)	0.98
Second eye (14)	0.33 (2.53)	-2.54 (1.75)	2.87 (0.82)	2.08	1.32 (0.47 to 2.09)	0.20
Without comorbidity (34)	0.30 (1.76)	-2.58 (2.20)	2.88 (0.48)	5.9	1.45 (0.90 to 1.96)	0.80
With comorbidity (40)	0.34 (1.64)	-2.89 (2.15)	3.23 (0.43)	7.5	1.69 (1.16 to 2.18)	0.97

# Independent-samples t test, p<0.001 for all except second eye group p = 0.002

SD = standard deviation and CI = confidence interval

*higher negative score indicates better visual function

The Catquest-8SF demonstrated a very high responsiveness to cataract surgery (effect size >1.00) for all the groups (Table 5). At baseline, the both eyes surgery group had the worst Catquest-8SF score and observed the maximum gain post-operatively and the highest effect size to cataract surgery than any other groups (Table 5). Except for the second-eye group, post-hoc power estimate was 80% or above. The groups with and without ocular co-morbidity had similar baseline scores. However, the group with co-morbidity had higher gain in the score and also demonstrated a higher effect size (Table 5). Except for the overall group, all other subgroups had 95% CI around effect size was greater than 1.00.

### Ready-to-use scoring spreadsheet

To ease the use of the 8SF-CN, a ready-to-use Microsoft Excel spreadsheets were hereby developed, which can be used to convert raw data to Rasch-scaled scores so that investigators could estimate person scores in logits directly without doing Rasch analysis when the study sample is similar to the present study (S1 File). More specifically, the spreadsheet consists of three sheets labeled as ‘rawdata’, ‘raschscore’, and ‘raw to Rasch conversion’. Once the investigators register patients’ responses to items in numerical label (i.e. 0 to 4) in the ‘rawdata’ sheet, a corresponding Rasch scores in the ‘raw to rasch conversion’ sheet could be easily obtained.

## Discussion

This study shows that the Chinese version of the Catquest-8SF-CN is psychometrically robust, valid, reliable and highly responsive to measure treatment effect in a Chinese population with cataract. Among the many cataract-specific exiting PRO instruments, the Catquest-9SF was reported to possess excellent psychometric properties when assessed by Rasch analysis.[15,19,20,22,39]. Our study has again reinforced the findings of these previous studies that the short-form Catquest is a high quality cataract-specific instrument in terms of its Rasch-based psychometric properties and responsiveness to capture treatment effect. [15,19,20,22,39].

The Catquest-9SF has been touted as one of the best quality and highly responsive outcome measure for cataract surgery.[10,15,18,19,22,40] Moreover, it has a few items (i.e., relatively a short instrument) and therefore, it has a high potential to be used as a routine clinical tool to assess visual function in a hospital setting. Thus, we purposefully selected the Catquest-9SF among the plethora of other existing cataract-specific PRO instruments. The broader aim of this study was to translate, culturally adapt, and revalidate it in our setting to explore whether it functions the same as in other populations. We used a meticulous multi-staged translation and cultural adaptation of the item content using a standard guideline to ensure that all the items of the Catquest are relevant and representative of Chinese socio-cultural status. This process has ensured face and content validity of the Chinese version of the Catquest. The subsequent validity tests (Rasch-based ISI/ISR and correlation with visual acuity) further provided the evidence of a strong validity of the Catquest-8SF-CN. Our study has also demonstrated that the Chinese Catquest is better targeted to Chinese people with cataract than in other studies. [15,22,39] This suggests that that the Catquest items are relevant and perfectly match to capture the impact of cataract in Chinese population.

The measurement precision of the original Catquest 9SF was good, indicating that the overall scale was able to discriminate at least three levels (or strata) of the participant abilities. One misfitting item was observed (item 7: Seeing to do delicate work). The phrase “delicate work” in item 7 is ambiguous in Chinese language. This might be one of the reasons that contributed to the item’s misfit. After the removal of item 7, precision of the 8-item Catquest, though slightly decreased, remained higher than acceptable value forming a psychometrically robust unidimensional scale. Similar to the previous studies in western countries, our study also found that the Catquest-8SF-CN was highly responsive to cataract surgery.[15,19,22] Moreover, the change in post-surgical scores and the ES were found to be much higher in this study than in the previous studies except for the second-eye surgery group.[10,19,22] This is probably because the Catquest is well targeted to our study population. The effect size in the second-eye surgery group was also high but it was the lowest among all other groups. The post-hoc power calculation showed that the estimate for the second-eye surgery group did not have the sufficient power to confirm the findings. A future study with a larger sample size in this group and the analysis with an adequate statistical power will confirm our findings.

The Chinese-8SF version demonstrated an excellent targeting compared to other similar PRO instruments, such as Activities of Daily Vision Scale (ADVS), Cataract Symptom Scale (CSS), Visual Disability Assessment (VDA) etc.[14,23,41] Nevertheless, these instruments suffered from poor targeting and serious ceiling and floor effects.[23] For instance, the CSS, an early-developed scale in 1990s, suffered a serious floor effect implying that the items were too easy for the ability of the patients.[41] While the present 8SF inherited the advantages of 9SF, the targeting was excellent, and no obvious ceiling or floor effect was observed (Table 3). [10, 19, 21] Moreover, the Catquest 8SF-CN is better targeted to Chinese cataract population than in other studies conducted in the western countries. This could be owing to a meticulous translation and careful cultural adaption to reflect the present state of the Chinese society and culture which ensures that instrument is more sensitive to measure the impact of the disease in the Chinese cataract population. Such superior metric properties were also found in our previous study of the Chinese version of the Visual Function Questionnaire (VF-8R) and the modified versions of the National Eye-Institute Visual Function Questionnaire (NEI VFQ).[11,12]

Our study has shown that people with bilateral cataract benefit the most from undergoing cataract surgery on both eyes together (Table 5). Similar, findings was reported in Canada when assessed with the Visual Function (VF-14) questionnaire.[42] Both-eyes surgery would allow rapid rehabilitation, lower cost and avoid sub-optimal visual function in daily life until the second-eye surgery.[43] In the country like China where there is an inadequate availability of cataract surgical services and the majority of the population live in rural areas. Both eyes cataract surgery for those with bilateral cataract would render more benefits to the patients and also improve effectiveness and efficiency by lowering direct and indirect costs of cataract.

The psychometric properties of the Catquest-9SF have been previously assessed in a Chinese population by Lin et al.[20] Our study further verifies the findings of Lin et al by showing that the Catquest is a valid, unidimensional and psychometrically robust measure of visual function in Chinese population with Cataract. Despite similar demographics between the study samples, we found that the Catquest was better targeted to our sample than in Lin et al study. Lin et al also reported that 2 items had DIF in their study sample; however we did not find any item biasness in our sample. One has to be noted that Lin et al study was limited to psychometric testing of the Catquest only. Our study further Lin et al’s message that the Catquest is not only a valid but also a very responsive tool to assess cataract surgery outcomes.

The main limitation of this study was a low follow-up rate and this probably adds to potential bias to our findings. This is probably because the majorities of the patients were relatively older and live far away from Wenzhou. Similarly, it is unlikely that those who are generally happy with the surgical outcome would travel a long distance to come for follow-up at the hospital unless there are complications. Generally, older Chinese patients do not like to come back to hospital again[7,44,45]. Further, only a small number (n = 14) of participants who followed-up had undergone second eye surgery. All the second eye surgery patients did the first surgery at least 6 months earlier from their current visit. As these patients attended our hospital for the second eye surgery, there might be a possibility of an inherent biasness by under-reporting their visual function at the baseline. These patients did their first cataract surgery in other hospitals therefore we did not have data to assess the cause and effect of the first eye surgery on the second eye surgery outcomes. A future study that takes in account of these issues in a larger sample size may unravel the exact influence of first eye surgery and timing on the second eye cataract surgery outcomes. The other limitation was that that only the Catquest was administered to the participants. Therefore, we could not assess convergent validity of the Catquest against the other PRO instrument/s.

The development of technologically advanced and smarter PRO instruments for the 21^st^ century to assess comprehensive ophthalmic quality of life is on the way in the form of item banks (The Eye-tem Bank Project). The item banks will be administered via computer adaptive testing (CAT) system and will be available through an online portal to researchers and clinicians around the world.[46] The item banks consist of Rasch calibrated refined items collected from different extant instruments (such as the Catquest-9SF and the VF-14) and the anew items supplemented via patients’ consultation. Our research group is currently developing such modern PRO instrument for all eye diseases across all populations and the project is named the Eye-tem Bank project,[47–51] which is funded by National Health and Medical Research Council (NHMRC) in Australia. The Eye-tem Bank will also be translated, adapted and validated in China. We call on researchers from around the world to join us to develop the Eye-tem Bank project to make it relevant across all the populations worldwide.

## Conclusions

The Catquest-8SF is a psychometrically robust, valid and highly responsive cataract-specific instrument in Chinese population with cataract. We have also demonstrated that cataract surgery has positive impact on people’s lives in terms of significantly improved patient-reported outcome measure after cataract surgery in China. This may be a testimonial of the real-world benefit people are experiencing after cataract surgery. Due to its shorter length and high responsiveness of the Catquest-8SF-CN, it has a high potential to be integrated into the routine cataract assessment in China.

## Supporting Information

S1 File The Catquest-8SF-CN- Raw to Rasch conversion. (XLS)
